# Identification of effective natural PIK3CA H1047R inhibitors by computational study

**DOI:** 10.18632/aging.203409

**Published:** 2021-08-20

**Authors:** Naimeng Liu, Xinhui Wang, Xuan Li, Xiaye Lv, Haoqun Xie, Zhen Guo, Jing Wang, Gaojing Dou, Ye Du, Dong Song

**Affiliations:** 1 Department of Breast Surgery, The First Hospital of Jilin University, Changchun, China; 2 Department of Oncology, First People’s Hospital of Xinxiang, Xinxiang, China; 3 Department of Obstetrics and Gynecology, Tangdu Hospital, The Fourth Military Medical University, Xi’an, China; 4 Department of Hematology, Honghui Hospital, Xi’an Jiao Tong University, Xi’an, China; 5 Clinical College, Jilin University, Changchun, China

**Keywords:** PIK3CA H1047R, drug development, virtual screening, TNBC, targeted therapy

## Abstract

Triple-negative breast cancer (TNBC) is a highly aggressive subtype of breast cancer with a poor prognosis and a high recurrence rate. *PIK3CA* gene is frequently mutated in breast cancer, with *PIK3CA H1047R* as the hotspot mutation reported in TNBC. We used the ZINC database to screen natural compounds that could be structurally modified to develop drugs targeting the PIK3CA H1047R mutant protein in the PI3K pathway. The LibDock module showed that 2,749 compounds could strongly bind to the PIK3CA H1047R protein. Ultimately, the top 20 natural ligands with high LibDock scores were used for further analyses including assessment of ADME (absorption, distribution, metabolism, and excretion), toxicity, stability, and binding affinity. ZINC000004098448 and ZINC000014715656 were selected as the safest drug candidates with strong binding affinity to PIK3CA H1047R, no hepatotoxicity, less carcinogenicity, better plasma protein binding (PPB) properties, and enhanced intestinal permeability and absorption than the two reference drugs, PKI-402 and wortmannin. Moreover, their lower potential energies than those of PIK3CA H1047R confirmed the stability of the ligand-receptor complex under physiological conditions. ZINC000004098448 and ZINC000014715656 are thus safe and stable leads for designing drugs against PIK3CA H1047R as part of a targeted therapeutic approach for patients with TNBC.

## INTRODUCTION

Triple-negative breast cancer (TNBC) is a highly aggressive molecular subtype of breast cancer, characterized by the absence of estrogen receptor (ER), progesterone receptor (PR), and human epidermal growth factor receptor 2 (HER2). According to the World Health Organization’s latest global cancer data, breast cancer has replaced lung cancer as the most common cancer worldwide. With a high recurrence rate and poor prognosis, TNBC accounts for nearly 10% to 20% of the new breast cancer cases worldwide [1–3]. Moreover, its 4-year survival rate is only 77%, despite intensive treatment strategies such as surgery and chemotherapy [4]. This highlights the need to develop new and effective treatment strategies.

The phosphoinositide-3 kinase (PI3K) pathway interacts with multiple pathways to perform numerous biological functions including proliferation, apoptosis, and metabolism. It is known to be frequently altered in human cancers, especially in breast cancer [5], with its overexpression associated with the activation of oncogenes such as *PIK3CA* and *MTOR* and inactivation of tumor suppressor genes such as *PIK3R1* and *PTEN* [6]. Of these, *PIK3CA* mutation is the most common and independent event in breast cancer. According to the Cancer Genome Atlas (TCGA) and the Catalogue of Somatic Mutations in Cancer (COSMIC), the mutation rate of the *PIK3CA* gene is 36% in breast cancer [7]. A study by Jiang et al. reported *PIK3CA* as the most frequently mutated gene (18%) in refractory TNBC [8], indicating its association with poor outcome and drug resistance [9].

*PIK3CA H1047R*, the hotspot mutation in TNBC, maps to the kinase domain of the protein [10]. PIK3CA H1047R expression induces dedifferentiation of lineage-restricted epithelial cells into a multipotent stem-like state, which further stimulates the development of heterogeneous mixed-lineage tumors [11]. In addition, certain studies have reported that TNBC with a *PIK3CA H1047R* mutation is less likely to result in a pathologic complete response (pCR) following chemotherapy [12].

With increasing chemotherapeutic resistance and poor prognosis in patients with TNBC, selective inhibitors of *H1047R* mutated protein can provide more effective treatment. For example, PKI-402 (ZINC000049745945), a highly specific PI3K inhibitor, is known to hinder breast cancer cell proliferation and block metastasis [13] by suppressing the phosphorylation of PI3K, particularly phosphorylated Akt (p-Akt) at T308 [14]. Wortmannin (ZINC000001619592), another PI3K inhibitor, can kill tumor cells by inducing double-stranded DNA breaks. Computational studies have shown a strong binding affinity of wortmannin with H1047R protein [15, 16]. However, these drugs have certain limitations, for example, PKI-402 is effective only at a high dose and the tumor may reoccur shortly after the treatment [14].

Recent years have witnessed a surge in the use of several natural products as leads that can be structurally modified to develop new and effective drugs [17]. We assessed the inhibitory effect of two natural compounds and their potential for drug development against PIK3CA mutated TNBC using computational methods. In addition, we compared the absorption, distribution, metabolism, excretion (ADME) and toxicity of these compounds with those of PKI-402 and wortmannin using predictive analysis.

## RESULTS

### Virtual screening of natural product database against PIK3CA H1047R

**Figure 1:**
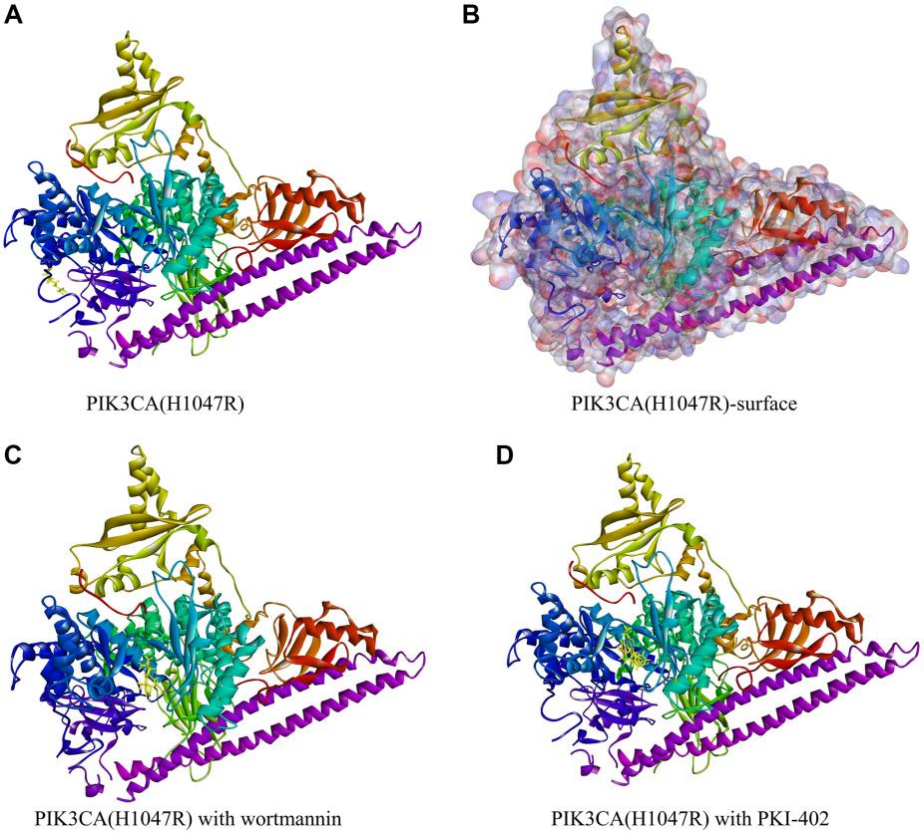
**Molecular structure of PIK3CA H1047R with the highlighted 1047Arg.** (**A**) The initial molecular structure. (**B**) Structure after binding area surface was added. Blue represents positive charge and red represents negative charge. (**C**) The molecular structure of PIK3CA H1047R with wortmannin. (**D**) The molecular structure of PIK3CA H1047R with PKI-402.

A total of 17,931 ligands were downloaded from the ZINC database. The chemical structure of PIK3CA H1047R (3HHM) was selected as the receptor to align these ligands. Of these, 2,794 compounds exhibited strong binding to PIK3CA H1047R protein. The top 20 ligands are listed in Table 1. PKI-402 (ZINC000049745945) and wortmannin (ZINC000001619592) were selected as two reference proteins (Figure 1).

**Table 1:** The top 20 ranked compounds with LibDock scores.

**Number**	**Compounds**	**LIBDOCK score**
1	ZINC000014780951	132.446
2	ZINC000014763060	131.304
3	ZINC000100168592	119.583
4	ZINC000004098466	119.396
5	ZINC000085808802	118.322
6	ZINC000000899675	118.184
7	ZINC000004098004	117.968
8	ZINC000085808820	117.475
9	ZINC000014715656	117.471
10	ZINC000013378578	117.305
11	ZINC000001714287	117.107
12	ZINC000044417879	116.723
13	ZINC000006094124	114.710
14	ZINC000004098448	114.209
15	ZINC000001667453	114.109
16	ZINC000014819753	112.782
17	ZINC000014820552	112.700
18	ZINC000008234257	112.644
19	ZINC000000388657	112.412
20	ZINC000032840897	112.136

### Absorption, distribution, metabolism, and excretion and toxicity analyses

We used the ADME module of the Discovery Studio 4.5 software to study the pharmacologic properties of the top 20 selected compounds, PKI-402, and wortmannin (Table 2). Except for ZINC000044417879, the aqueous solubility of all compounds was predicted to be high. Half of the top 20 compounds had undefined blood–brain barrier (BBB) permeability, whereas others had medium or low permeability, except for ZINC000001714287 and ZINC000004098004. Nearly all compounds including the two targeted drugs effectively inhibited cytochrome P450 2D6 (CYP2D6), except for ZINC000032840897 and ZINC000004098448. Eleven compounds and PKI-402 did not exhibit hepatotoxicity, whereas 9 compounds and wortmannin showed an opposite effect. We next predicted the extent of intestinal permeability and drug absorption of these compounds. ZINC000044417879 and ZINC000008234257 showed the worst and poor intestinal permeability, respectively, and others exhibited a good level of permeability and absorption, except for PKI-402, ZINC000100168592, ZINC000004098466, and ZINC000032840897. An analysis of plasma protein-binding (PPB) properties predicted that 6 compounds had weak binding, similar to that of wortmannin and PKI-402.

**Table 2:** Adsorption, distribution, metabolism, and excretion properties of compounds.

**Number**	**Compounds**	**Solubility level**	**BBB level**	**CYP2D6**	**Hepatotoxicity**	**Absorption level**	**PPB level**
1	ZINC000100168592	3	4	1	1	1	0
2	ZINC000014819753	2	2	1	0	0	0
3	ZINC000006094124	3	3	1	0	0	0
4	ZINC000004098004	2	1	1	0	0	0
5	ZINC000004098466	2	4	1	1	1	0
6	ZINC000014820552	2	2	1	0	0	0
7	ZINC000014780951	3	4	1	1	0	1
8	ZINC000008234257	3	4	1	1	2	1
9	ZINC000004098448	2	2	0	0	0	0
10	ZINC000013378578	3	4	1	0	0	1
11	ZINC000000388657	3	2	1	0	0	0
12	ZINC000001667453	3	4	1	1	0	0
13	ZINC000044417879	1	4	1	1	3	0
14	ZINC000032840897	3	4	0	1	1	1
15	ZINC000001714287	2	1	1	0	0	0
16	ZINC000014763060	2	4	1	0	0	0
17	ZINC000014715656	2	2	1	0	0	0
18	ZINC000000899675	3	4	1	0	0	0
19	ZINC000085808820	4	3	1	1	0	1
20	ZINC000085808802	4	3	1	1	0	1
21	wortmannin	2	3	1	1	0	1
22	PKI-402	3	4	1	0	1	1

Abbreviations: BBB: blood-brain barrier; CYP2D6: cytochrome P-450 2D6; PPB: plasma protein binding.

Aqueous-solubility level: 0, extremely low; 1, very low, but possible; 2, low; 3, good.

BBB level: 0, very high penetrant; 1, high; 2, medium; 3, low; 4, undefined.

CYP2D6 level: 0, noninhibitor; 1, inhibitor.

Hepatotoxicity: 0, nontoxic; 1, toxic.

Human-intestinal absorption level: 0, good; 1, moderate; 2, poor; 3, very poor.

PPB: 0, absorbent weak; 1, absorbent strong.

We next screened the drugs for safety using the TOPKAT module based on the two-dimensional (2D) structures of 20 compounds and two reference drugs. The toxic effects of these compounds on humans and the environment were assessed using Ames mutagenicity test (Ames test), the developmental toxicity potential (DTP) test, and the U.S. National Toxicology Program (NTP) database rodent carcinogenicity test (Table 3). Twelve compounds were found to be non-mutagenic; in addition, 10 compounds in male mice, 14 compounds in female rats, and 9 compounds in male rats were non-carcinogenic. On the contrary, both PKI-402 and wortmannin were found to be carcinogenic in these three groups of animals.

**Table 3:** Toxic effects of compounds.

**Number**	**Compounds**	**Mouse NTP**		**Rat NTP**		**Ames**	**DTP**
		**Female**	**Male**	**Female**	**Male**		
1	ZINC000100168592	0	0.999	0	0	1	0
2	ZINC000014819753	1	1	0.998	0.929	0	1
3	ZINC000006094124	0	0	1	0.068	0	0.881
4	ZINC000004098004	0	1	0	0.127	0	0.998
5	ZINC000004098466	0.09	1	1	1	1	1
6	ZINC000014820552	0	0	1	0.63	0	0.997
7	ZINC000014780951	0.982	1	0	1	0	0.016
8	ZINC000008234257	1	0.705	0	0.373	0	1
9	ZINC000004098448	0	0	0	0	0.753	1
10	ZINC000013378578	0	1	0	0.974	0.046	0
11	ZINC000000388657	0.677	0	0.017	0.009	0.989	0.986
12	ZINC000001667453	0.868	0	0.942	0.992	0.706	1
13	ZINC000044417879	0.001	0	0	0	0	1
14	ZINC000032840897	0	0.045	0	0	0.435	0
15	ZINC000001714287	0.927	0.405	1	0	0	1
16	ZINC000014763060	0.999	1	0	0.994	0	1
17	ZINC000014715656	0	0.095	0	0	1	1
18	ZINC000000899675	0	1	0	1	0.444	1
19	ZINC000085808820	0	0.02	0	0.946	0	0
20	ZINC000085808802	0	0.02	0	0.946	0	0
21	wortmannin	0.054	1	1	1	0	0
22	PKI-402	0	1	0.988	0.902	0.044	0

Abbreviations: NTP: U.S. National Toxicology Program; DTP: developmental toxicity potential. NTP < 0.3 (noncarcinogen); > 0.8 (carcinogen). Ames < 0.3 (non-mutagen); > 0.8 (mutagen). DTP < 0.3 (nontoxic); > 0.8 (toxic).

**Figure 2:**
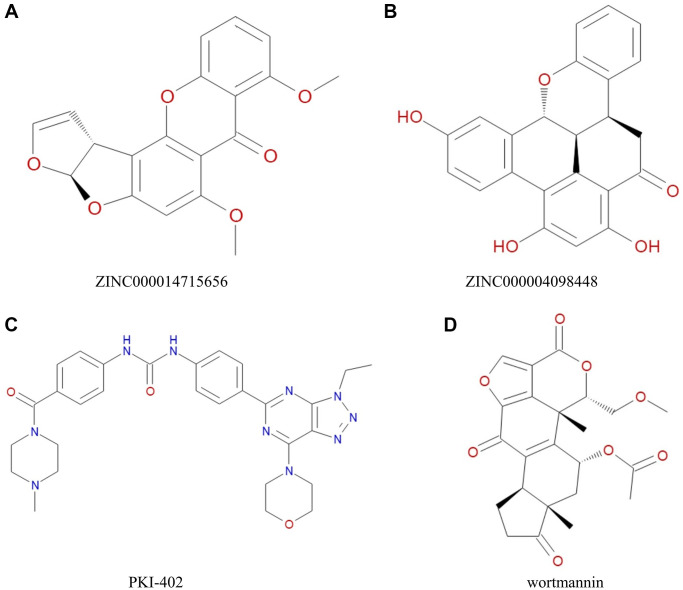
Chemical structure of (**A**) ZINC000014715656 (**B**) ZINC000004098448 (**C**) PKI-402 (**D**) Wortmannin.

These results showed ZINC000004098448 and ZINC000014715656 as potential candidate drugs with no hepatotoxicity, less carcinogenicity, better PPB properties, and enhanced intestinal permeability and absorption than other compounds and two reference drugs. Thus, ZINC000004098448 and ZINC000014715656 were selected as safe lead compounds for further studies (Figure 2).

### Analysis of ligand binding and ligand pharmacophore

**Figure 3:**
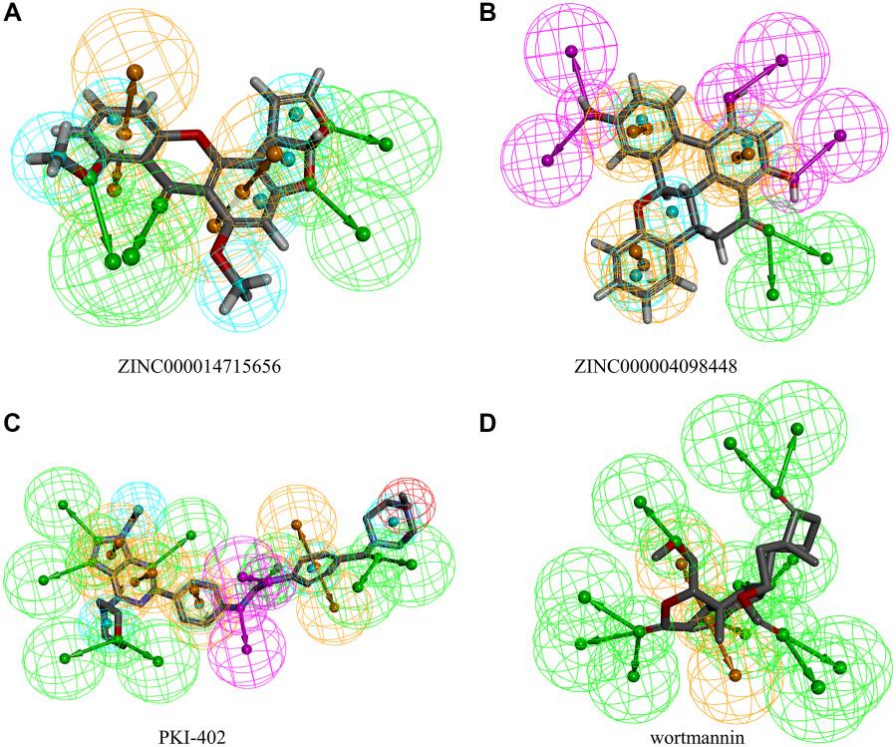
Pharmacophores of (**A**) ZINC000014715656 (**B**) ZINC000004098448 (**C**) PKI-402 (**D**) Wortmannin.

The pharmacophore study of the two candidate ligands, PKI-402, and wortmannin showed 14 features in ZINC000014715656 including 4 hydrogen bond (HB) acceptors, 6 hydrophobics, and 4 ring aromatics (Figure 3). ZINC000004098448 showed 20 features in ZINC000004098448, including 6 HB acceptors, 4 HB donors, 4 hydrophobics, and 6 ring aromatics. Wortmannin and PKI-402 showed 13 and 24 features, respectively.

We used the CHARMM force field-based CDOCKER method to study the binding affinity and mechanism between these compounds and 3HHM. ZINC000004098448 and ZINC000014715656 bound to 3HHM in the CDOCKER module served as the reference drugs; their potential binding energies are listed in Table 4. The CDOCKER potential energy of ZINC000014715656 (–43.6257 kcal/mol) was close to that of wortmannin (–41.5097 kcal/mol) and PKI-402 (–42.9796 kcal/mol). In addition, the CDOCKER potential energy of ZINC000004098448 (–51.3249 kcal/mol) was highly lower than those of reference drugs, showing the strong binding of 3HHM with ZINC000004098448 and ZINC000014715656 than PKI-402 and wortmannin.

**Table 4:** CDOCKER potential energy of compounds with PIK3CA H1047R.

**Compounds**	**-CDOCKER potential energy (kcal/mol)**
ZINC000014715656	43.6257
ZINC000004098448	51.3249
wortmannin	41.5097
PIK-402	42.9796

**Figure 4:**
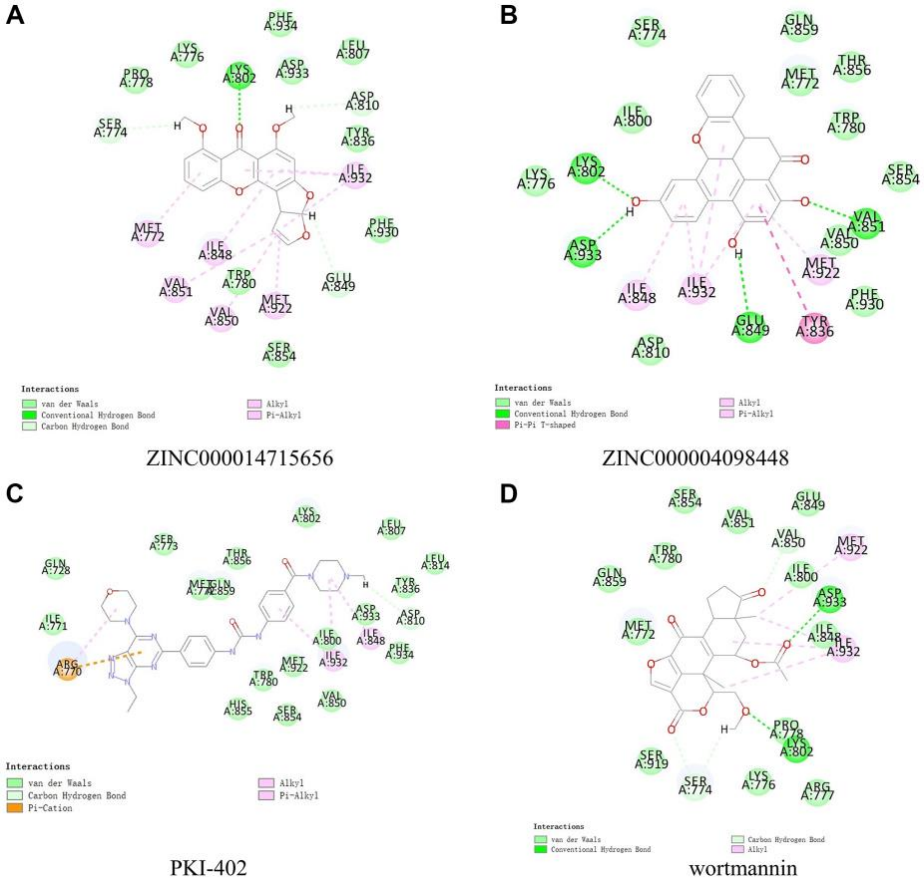
Schematic representation showing intermolecular interactions of predicted binding modes of (**A**) ZINC000014715656 with PIK3CA H1047R, (**B**) ZINC000004098448 with PIK3CA H1047R, (**C**) PKI-402 with PIK3CA H1047R, and (**D**) wortmannin with PIK3CA H1047R.

**Figure 5:**
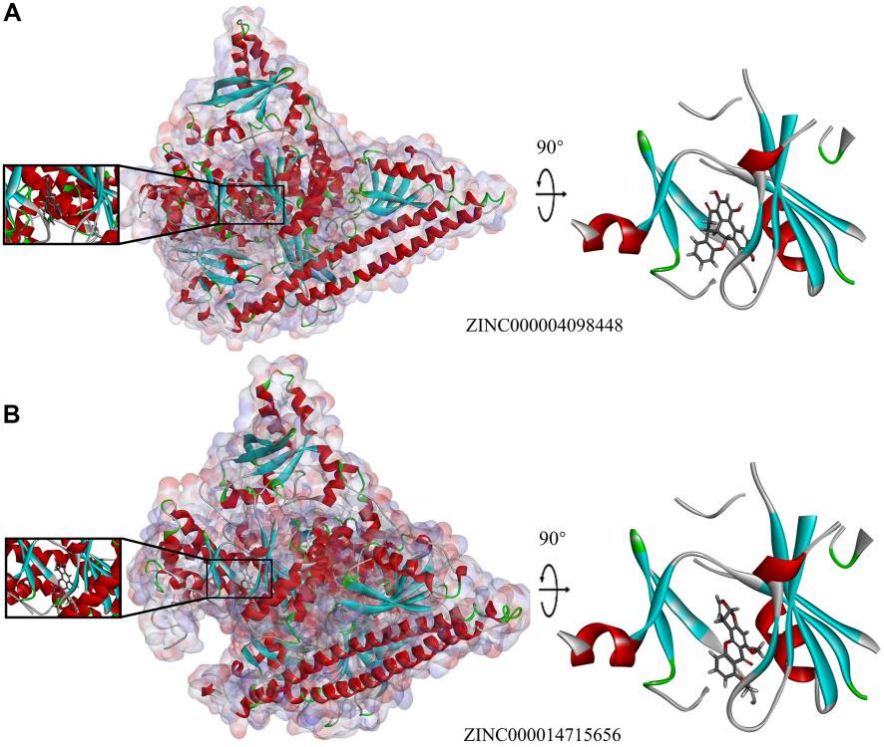
**Schematic representation showing interactions between ligands and 3HHM.** The binding area surface was added. Blue represents positive charge and red represents negative charge. Ligands are shown in sticks, with the structure around the ligand–receptor junction shown in thinner sticks. (**A**) ZINC000004098448–3HHM complex. (**B**) ZINC000014715656–3HHM complex.

**Figure 6:**
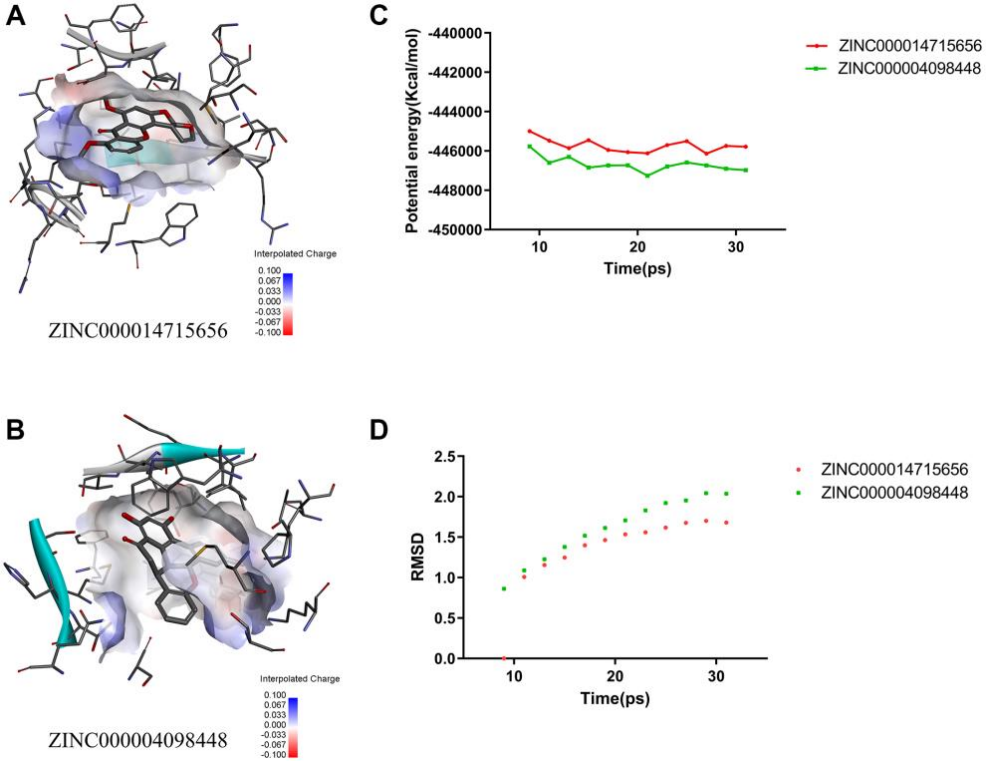
(**A**) The charge between the ZINC000014715656–3HHM surface. (**B**) The charge between the ZINC000004098448–3HHM surface. (**C**) Potential energy of ZINC000004098448 and ZINC000014715656, with average root-mean-square deviation. (**D**) RMSD of ZINC000004098448 and ZINC000014715656.

Next, we conducted structural computational studies to obtain hydrogen bonds, π-related interactions, and the charge on these ligands (Figures 4, 5 and 6). ZINC000014715656 formed 4 pairs of hydrogen bonds (Table 5) between O10 of the compound and LYS802:HZ2 of 3HHM, H26 of the compound and SER774:OG of 3HHM, H36 of the compound and ASP810:OD1 of 3HHM, and H36 of the compound and GLU849:O of 3HHM. Eight pairs of π-related interactions are listed in Table 6. In addition, ZINC000004098448 formed 4 pairs of hydrogen bonds between LYS802:HZ2 of 3HHM and O16 of the ligand, VAL851:HN of 3HHM and O25 of the ligand, H38 of the ligand and ASP933:OD1 of 3HHM, and H41 of the ligand and GLU849:O of 3HHM. Six pairs of π-related interactions, including TYR836, ILE932, ILE848, ILE932, MET922, and ILE932 of 3HHM were found with ZINC000004098448. Wortmannin exhibited 6 hydrogen bonds with 3HHM and 4 π-related interactions with 3HHM, whereas PKI-402 displayed 1 hydrogen bond with 3HHM and 4 π-related interactions with 3HHM (Tables 5 and 6).

**Table 5:** Hydrogen bond interaction parameters of each compound with PIK3CA H1047R.

**Receptor**	**Compound**	**Donor atom**	**Receptor atom**	**Distances (Å)**
3HHM	ZINC000014715656	LYS802:HZ2	ZINC000014715656:O10	1.94
		ZINC000014715656:H26	SER774:OG	3.05
		ZINC000014715656:H32	ASP810:OD1	3.03
		ZINC000014715656:H36	GLU849:O	2.08
	ZINC000004098448	LYS802:HZ2	ZINC000004098448:O16	1.86
		VAL851:HN	ZINC000004098448:O25	2.12
		ZINC000004098448:H38	ASP933:OD1	2.11
		ZINC000004098448:H41	GLU849:O	2.84
	wortmannin	LYS802:HZ1	Molecule:O2	2.15
		ASP933:HN	Molecule:O31	2.38
		SER774:HB2	Molecule:O7	2.77
		VAL850:HA	Molecule:O23	2.29
		ASP933:HA	Molecule:O31	3.07
		Molecule:H34	SER774:OG	2.54
	PKI-402	Molecule:H60	ASP810:OD1	2.69

**Table 6:** π-Related interaction parameters of each compound with PIK3CA H1047R.

**Receptor**	**Compound**	**Donor atom**	**Receptor atom**	**Distances (Å)**
3HHM	ZINC000014715656	VAL850	ZINC000014715656	5.10
		VAL851	ZINC000014715656	5.45
		ZINC000014715656	MET922	4.85
		ZINC000014715656	ILE932	5.27
		ZINC000014715656	MET772	5.30
		ZINC000014715656	ILE932	5.14
		ZINC000014715656	ILE848	4.86
		ZINC000014715656	ILE932	4.25
	ZINC000004098448	TYR836	ZINC000004098448	5.83
		ILE932	ZINC000004098448	5.30
		ZINC000004098448	ILE848	4.78
		ZINC000004098448	ILE932	5.02
		ZINC000004098448	MET922	5.33
		ZINC000004098448	ILE932	4.63
	wortmannin	ILE932	Molecule	4.93
		Molecule:C14	ILE932	4.36
		Molecule:C25	MET922	4.42
		Molecule:C25	ILE932	3.81
	PKI-402	ARG770	Molecule	5.37
		ILE848	Molecule	4.37
		ILE932	Molecule	5.22
		Molecule	ILE932	5.23

### Molecular dynamics simulation

We next used a molecular dynamics simulation module to study the stability of ligand–3HHM complexes under natural circumstances. Molecular docking experiments were conducted to evaluate the potential binding energies of these complexes and obtain the root mean square deviation (RMSD) curves (Figure 6). The trajectories of the two complexes reached the equilibrium after 30 ps and gradually stabilized with time. The hydrogen bonding and π-related interactions between the compounds and 3HHM contributed to the stability of these complexes. In conclusion, ZINC000004098448 and ZINC000014715656 could stably bind with 3HHM under natural circumstances.

## DISCUSSION

Triple-negative breast cancer is the most aggressive and highly invasive molecular subtype of breast cancer with a high recurrence rate and low median survival [4, 18]. The PI3K/AKT pathway is one of the most frequently altered pathways in TNBC [19]. The hyperactivation of the PI3K/AKT pathway due to mutations in *PIK3CA* gene results in cell cycle dysregulation and consequently tumorigenesis [20–22]. Among these, *PIK3CA H1047R* is a hotspot mutation that maps to the kinase domain of the protein [10].

Targeted drug therapy along with radiotherapy has emerged as a recent therapeutic approach to treat different subtypes of breast cancer. Although numerous drugs are available against TNBC, only a few, such as PKI-402 and wortmannin, are known to target PIK3CA H1047R. In addition, shortcomings such as the high dose requirement of PKI-402 and recurrence within a short interval, limit their clinical applications [14].

We used different modules of the Discovery Studio 4.5 software to screen potential natural ligands for drug development. After 17,931 natural ligands were downloaded from the ZINC 15 database, the LibDock module showed that 2,749 compounds could strongly bind with PIK3CA H1047R. Finally, the top 20 natural ligands with high LibDock scores were used for further analyses.

The ADME and TOPKAT modules were used to predict and evaluate the pharmacologic properties of these 20 compounds. Two natural ligands, namely ZINC000004098448 and ZINC000014715656, were found to be non-hepatotoxic, water-soluble, and fulfilling the ADME criteria. The TOPKAT results showed that these two compounds had less carcinogenicity and could safely replace PKI-402 and wortmannin as targeted drugs.

The CDOCKER module, used to analyze the binding affinity and mechanism of ligands with 3HHM, revealed that ZINC000004098448 had lower potential energy than PKI-402 and wortmannin, whereas the potential energy of ZINC000014715656 was similar to those of reference drugs. Structural computational studies showed the formation of hydrogen bonds and π-related interactions by lead compounds and reference drugs. The results indicated that ZINC000004098448 and ZINC000014715656 had a higher binding affinity for 3HHM than PKI-402 and wortmannin.

Finally, a molecular dynamics simulation model was used to calculate RMSD and potential energy and assess the stability of compound–3HHM complexes. The trajectories of both ZINC000004098448–3HHM and ZINC000014715656–3HHM complexes reached the equilibrium after 30 ps and gradually stabilized with time, implying the stability of two complexes under physiological conditions. These findings indicated that ZINC000004098448 and ZINC000014715656 could be used to develop targeted therapy for patients with TNBC harboring PIK3CA H1047R mutation.

## CONCLUSIONS

Compared to standard chemotherapy and surgery, molecular targeted therapies are more specific with lesser side effects. We conducted a comprehensive computational study to identify potential effective inhibitors of PIK3CA H1047R. Altogether, our results showed ZINC000004098448 and ZINC000014715656 as promising candidates to develop targeted therapy against PIK3CA mutated TNBC. However, further *in vivo* and *in vitro* studies are warranted before these drug candidates could enter the market for clinical applications.

## MATERIALS AND METHODS

### Discovery studio 4.5 software and ligand database

Discovery Studio 4.5 software is used to simulate the systems of small molecules and macromolecules. It provides easy-to-use and valuable tools for protein simulation, optimization, and drug design. Discovery Studio is a visualization tool for viewing and analyzing protein and modeling data by integrating the storage and management of experimental data using modeling and simulation tools.

The database of natural products was downloaded from the ZINC database and lead compounds were screened. The ZINC database is a freely available toolset designed by the Irwin and Shoichet Laboratories, Department of Pharmaceutical Chemistry, University of California, San Francisco that provides access to commercially available compounds for virtual screening and ligand and pharmacophore identification [23].

### Structure-based virtual screening using LibDock

We used LibDock to screen ligands with potential inhibitory activity against PIK3CA H1047R. The crystals of p110alpha H1047R mutant in complex with wortmannin (Protein Data Bank identifier: 3HHM), wortmannin inhibitor (Protein Data Bank identifier: ZINC000001619592), and PKI-402 inhibitor (Protein Data Bank identifier: ZINC000049745945) were downloaded from the ZINC database and the RCSB Protein Data Bank. The chemical structure of 3HHM is shown in Figure 2. Next, these protein complexes were imported to LibDock. For LibDock analysis, the proteins were prepared by removing the crystal water and other heteroatoms, followed by the addition of hydrogen and protonation, ionization, and energy minimization. The binding region of wortmannin to PIK3CA H1047R was selected as the binding site. The active site for docking was generated, and the LibDock scores of these compounds were ranked and listed [24].

### Absorption, distribution, metabolism, and excretion and toxicity prediction

We used the ADME and TOPKAT modules to calculate the absorption, distribution, metabolism, and excretion (ADME) and the toxicity of these compounds. In addition, the safety and pharmacologic properties of these compounds were studied during the selection of natural ligands for PIK3CA H1047R [25].

### CDOCKER for molecule docking and ligand pharmacophore prediction

Molecular docking was performed using the CDOCKER module, which is based on CHARMM force field. The compounds were prepared for docking by removing water molecules and adding hydrogen atoms to 3HHM protein because fixed water molecules can affect the conformation of the receptor–ligand complex [26].

Pharmacophore modeling was performed using feature mapping with predictive activity. This method uses a series of compounds with well-defined activity values for specific biological targets. Feature mapping can analyze five kinds of molecular patterns, including hydrophobic, hydrogen bond (HB) acceptor, HB donor, ring aromatic, and positive ion.

### Molecular dynamics simulation

The molecule docking program was used to select the best conformation of ligand–3HHM complexes. The complexes were imported to molecular dynamics simulation program. Sodium chloride was added to the system to simulate the physiological environment. The CHARMM field force was used for energy minimization. Next, the trajectory was determined for potential energy and structural characteristics of compounds using Discovery Studio 4.5.
